# Geropsychology Workforce Development: Mentoring and Outreach by the American Board of Geropsychology

**DOI:** 10.1093/geroni/igaa057.2000

**Published:** 2020-12-16

**Authors:** Michelle Mlinac, Heather Smith

**Affiliations:** 1 VA Boston Healthcare System, Jamaica Plain, Massachusetts, United States; 2 Milwaukee VA Medical Center, Milwaukee, Wisconsin, United States

## Abstract

To build workforce capacity and increase access to geropsychology services across the country, the American Board of Geropsychology (ABGERO) is engaged in efforts to promote competence in the specialty of Geropsychology. ABGERO developed a mentoring program to encourage psychologists to pursue board certification by demonstrating knowledge, skills, and abilities in delivering professional services to older adults. Mentors provide support around exam preparation, develop learning plans for psychologists new to the specialty, and help mentees consolidate their professional identities as geropsychologists. Candidates receiving mentorship include early career psychologists who completed geropsychology fellowships, mid-late career geropsychologists who seek board certification to be generative to the field, and psychologists looking to build expertise in geropsychology. For this latter group, clinical consultation groups were also created. Currently, 20 geropsychologists mentor 41 psychologists and 2 graduate students. Two geropsychologists have provided weekly consultation to 15 psychologists. Future implications for mentoring within geropsychology will be discussed.

